# EHMTI-0138. Significance of neurovascular contact in classical trigeminal neuralgia

**DOI:** 10.1186/1129-2377-15-S1-C39

**Published:** 2014-09-18

**Authors:** S Maarbjerg, F Wolfram, A Gozalov, J Olesen, L Bendtsen

**Affiliations:** 1 Danish Headache Center Department of Neurology, Glostrup Hospital University of Copenhagen, Glostrup, Denmark; 2 Department of Diagnostics, Glostrup Hospital University of Copenhagen, Glostrup, Denmark

## Introduction

Neurovascular contact (NVC) is considered a frequent cause of classical trigeminal neuralgia (TN) and transposition of a blood vessel in the potentially dangerous procedure of microvascular decompression is considered over other surgical options in medically refractory TN. However, prevalence of NVC has not been investigated in a representative neurological TN patient cohort using high-quality imaging and blinded evaluator.

## Aim

We aimed to investigate whether presence and degree of NVC are correlated to pain side in TN.

## Methods

Consecutive TN patients were referred to 3.0 Tesla MRI and included in a cross-sectional study. MRI scans were evaluated blindly and graded according to presence and degree of NVC. Severe NVC was defined as displacement or atrophy of the trigeminal nerve.

## Results

A total of 135 TN patients were included. NVC in general was prevalent on both symptomatic and asymptomatic side (89% vs. 78%, p = 0.014, OR = 2.4 (1.2-4.8), p = 0.017) while severe NVC was highly prevalent on symptomatic compared to asymptomatic side (53% vs. 13%, p < 0.001, OR = 11.6 (4.7-28.9), p < 0.001). Severe NVC was caused by arteries in 98%.

## Conclusions

Severe NVC is associated to pain side in TN, while any type of NVC is common on both symptomatic and asymptomatic side. Findings demonstrate importance of NVC grading and have important implications for understanding of TN etiology and most likely for patient selection for microvascular decompression.

No conflict of interest.

